# Research on the Creep Model of Deep Coal Roadway and Its Numerical Simulation Reproduction

**DOI:** 10.3390/ijerph192315920

**Published:** 2022-11-29

**Authors:** Qiming Zhang, Enyuan Wang, Zeng Ding

**Affiliations:** 1 Key Laboratory of Gas and Fire Control for Coal Mines (China University of Mining and Technology), Ministry of Education, Xuzhou 221116, China; 2 School of Safety Engineering, China University of Mining and Technology, Xuzhou 221116, China; 3 Geotechnical Institute, TU Bergakademie Freiberg, 09599 Freiberg, Germany

**Keywords:** deep coal roadway, fractional-order elements, nonlinear viscoelastic-plastic model, secondary development, numerical simulation

## Abstract

The long-term stability of coal mine roadway engineering is critical to the safe mining of coal resources and the protection of the surface environment. In this paper, the creep test of coal samples in coal roadway was carried out by multi-stage constant load method, and the test results showed that when the stress level was low, the creep curve had a attenuated stage and a steady-state stage, and the steady-state creep rate tended to increase with the increase in the stress level; When the stress level was higher than the yield stress, the creep rate curve appeared to have an acceleration stage after the steady-state stage. The instability failure mode of the coal sample was mainly shear failure with local tension failure. For this, a New Fractional-order Nonlinear Viscoelastic-plastic Rheological Model (NFNVRM) was established by introducing Abel elements and Nonlinear elements, and the constitutive equation of the model was deduced. The new model can fully reflect the stable decay stage and accelerated rheological stages of coal samples, and the parameter identification curve was consistent with the experimental results, which verifies the correctness and reasonableness of the NFNVRM. Meanwhile, based on the FLAC3D secondary development interface, the constitutive equations of the NFNVRM were written into the software to obtain new Dynamic Link Library (DLL) files. The simulation results were consistent with the experimental results when the DLL file was called. Finally, the NFNVRM.dll was applied to predict the surrounding rock deformation of an S mine. The study’s findings offer suggestions for environmental protection.

## 1. Introduction

Rheological behavior is a term used to describe a process in which the deformation of the medium grows over time while under constant external load, and may eventually lead to damage. Rheological phenomena are common in all kinds of geotechnical engineering, and the engineering problems caused by rheology, such as long-term stability and safety, are becoming increasingly prominent, which seriously affect the extraction of underground energy resources [1,2]. Especially for coal resources, with the reduction of shallow resources, many coal mines have begun to turn to deep resource mining. Tunnels and underground roadways under deep buried conditions are in a complex environment of “three highs and one disturbance”, which results in the contradiction among the surrounding rock characteristics, strength, and stress, where the roadway surrounding rock deformation and stability control problems occur frequently [3,4,5]. Therefore, there is an urgent need to understand the rheological properties of coal or rock for the construction of deep major rock projects, especially coal mine tunnel projects, in order to promote the successful extraction of underground resources and to deeply study the coal or rock rheological model that ensures the security and stability of engineering construction in the long-term operation process.

Currently, research work related to coal or rock rheology is progressing rapidly, and some achievements have been obtained in both experimental and theoretical aspects.

Rheological properties tests can visually reveal the rheological pattern of rocks, which has always been an important way to study the rheological properties of rocks. Kawada et al. [6] studied the general constitutive equations of the viscoelastic behavior of rocks and minerals with fractional derivatives, and proposed a method for determining the order of fractional derivatives of the viscoelastic behavior of rocks and minerals. Wang et al. [7] conducted triaxial creep tests on a coal body under a high stress state. The results showed that the axial and circumferential directions of the specimen showed obvious three-stage rheological phenomenon, and the creep deformation decreased with the increase in confining pressure. Zhang et al. [8] established a nonlinear viscoelastic plastic model through multi-stage rheological tests of a coal body and verified the model. Liu et al. [9] studied the rheological deformation of a soft rock specimen under the conditions of stress and water softening, explored the influence of stress and water on rock creep, and analyzed their mechanisms. Wang et al. [10] introduced the damage variable that considers the proportion of irrecoverable plastic strain to the total strain, and established a new nonlinear multivariate creep model by connecting the newly proposed damage viscous body with the Hooken substance, the Saint Venant body, and the Kelvin element in series. Tang et al. [11] studied the effect of water saturation on the rheomechanical behavior of red sandstone and concluded that saturated water increased the minimum creep strain rate of the rock and significantly reduced the failure time. Mansouri et al. [12] studied the creep properties of salt rocks under different gradient stresses, and based on creep curves and rock parameters, obtained a steady-state creep rate constitutive equation, established the relationship between the microscopic and macroscopic deformation of salt rocks, and derived the plastic constitutive equation of salt rocks, which can reflect the deformation mechanism of plasticity and the creep of salt rocks. Zhang et al. [13] studied the coupling effect of triaxial stress and gas pressure on coal, considered effective stress in the Nishihara model, and conducted an in-depth analysis of the creep stage of coal and rock. Numerous scholars from teams such as Liu et al. [14], Lin et al. [15], Wu et al. [16], Gutiérrez-Ch et al. [17], Lyu et al. [18], and Zhang et al. [19] have made more research results on uniaxial and triaxial compression creep in soft rocks such as tuffs, shales, siltstones, salts, weathered sandstones, and coal bodies.

The study of rock rheological models has been a hot topic in the study of rock rheology theory and a key issue in applying the theory to practical engineering. There are two main approaches to modelling the nonlinear rheology of rocks as follows: One is to use nonlinear rheological elements to replace conventional linear rheological elements to establish a rheological constitutive model that can describe the accelerating rheological stage of rocks; the second is to adopt new theories, such as internal time theory and fracture mechanics, to establish a rock rheological constitutive model. The rheological constitutive models developed by both methods can better describe the accelerated rheological stages of the rocks. In recent years, some nonlinear rheological models and theories have been developed in rock mechanics. Representative ones are as follows: Yang et al. [20] proposed a new non-stationary and nonlinear viscoelastic shear creep model based on the viscoelastic shear creep test results of shale with four different shear stress levels; Cong et al. [21] proposed a new nonlinear elastic-plastic rock rheological model based on experimental results combined with the nonlinear elastomeric plastic deformation characteristics of rocks. Bouras et al. [22] proposed a fractional viscoelastic creep model for high-temperature concrete, which can effectively reflect time-varying stress and temperature; Zhao et al. [23] performed a series of triaxial creep tests on fractured limestone specimens under multi-stage loading and unloading cycles and proposed a nonlinear elastomeric creep instanton model. Haghighat et al. [24] developed a viscoelastic model that reproduces the creep behavior and inelastic deformation of rocks during the loading– unloading cycle; Huang et al. [25] proposed a new constitutive model of soft rock creep in combination with disturbing stresses, and the model can effectively predict the deformation and damage mode of the tunnel with time. Since the nonlinear rheological component model of rocks helps to conceptually understand the elastic and plastic components of deformation, and the expressions often directly describe creep and relaxation, many researchers in rock mechanics use the nonlinear rheological component model to explain the various mechanical properties of rocks [26,27,28,29].

Relevant research on coal or rock rheological properties and rheological constitutive models are still difficult and hot issues. In contrast to rock, coal has significant elastic phases. In view of this, this paper conducted conventional triaxial creep test research on coal samples to analyze the influence of constant load on mechanical parameters and deformation; a NFNVRM of coal based on fractional calculus was proposed, and its nonlinear creep and relaxation characteristics were analyzed theoretically. Secondary development based on FLAC3D software (Itasca Consulting Group, Inc., Minneapolis, MN, USA) was also carried out to promote the application of rock nonlinear rheological modelling theory in engineering practice.

## 2. Experimental Investigation

### 2.1. Preparation and Properties of Sample

**Figure 1:**
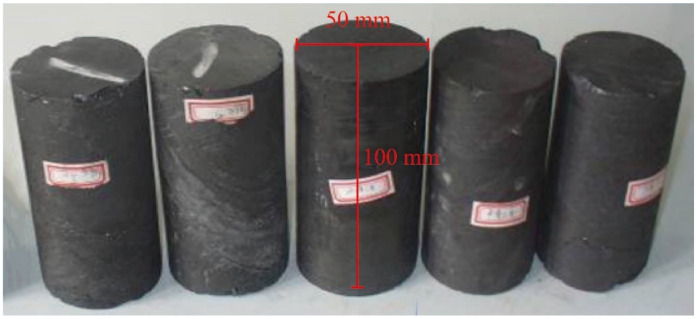
Picture of uniaxial compression sample.

The coal samples used in the test were taken from the surrounding rock of the deep coal roadway of a mine, and the buried depth was about 900 m. According to the ISRM test standard, the coal sample was made into a cylinder with a diameter of 50 mm and a height of 100 mm. In order to reduce the test error, the surface of the coal sample and the upper and lower ends were polished, and the dimensional error and unevenness were less than 0.5 mm, as show in Figure 1.

Through the uniaxial compression test of a complete coal sample, the experimental results are shown in Table 1 below. The average instantaneous compressive strength of the measured sample was about 9.98 MPa, and the long-term strength was 75% of the instantaneous compressive strength, which was about 7.5 MPa.

### 2.2. Test Equipment

**Figure 2:**
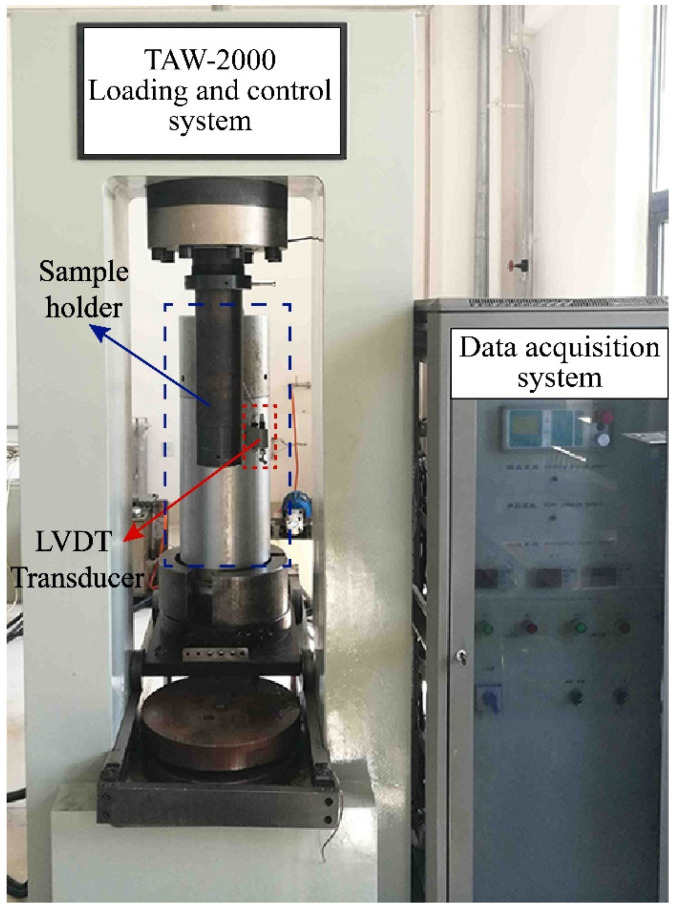
Experimental system diagram.

The test system included a conventional three-axis loading and control system, a data acquisition system, etc. (Figure 2). The loading control system was composed of a gripper, a high-precision plunger pump, a water bath, and a gas injection pipeline, which can provide maximum axial stress of 50 MPa, maximum circumferential stress of 40 MPa, a test force accuracy of ±1%, a test force resolution of 1/120,000, a displacement accuracy of ±1%, a displacement resolution of 1/100,000, and a deformation resolution of 1/100,000. The deformation measurement range was 0–10 mm in the axial direction and 0–5 mm in the radial direction. The creep loading system had a smooth loading process and good long-term stability. Within the range of axial test force, shear test force, and confining pressure, the fluctuation of 100 h force value was less than 1/100. The data acquisition system adopted microcomputer electro-hydraulic servo closed-loop control, which could arbitrarily set the loading rate, temperature, and gas injection rate. The experimental deformation data were automatically collected by a LVDT and strain collector, and the computer displayed the data curve in real time.

### 2.3. Test Plan

To avoid the influence of the external environment, the tests were conducted in a constant temperature and humidity rheological laboratory. The room temperature was controlled at (20 ± 3) °C and the humidity was controlled at 40%.

Since the coal samples are hardly identical in nature, it is difficult to achieve identical test conditions for constant loading tests. Besides, more specimens and longer time are required, and the data obtained are more discrete. The test was conducted in a stepped loading mode, and the load was applied step-by-step according to the instantaneous compressive strength. The stress of level 1 load is proposed to be 30–50% of the conventional compressive strength, about 5 MPa, and the applied load is about 9.8 kN. The following levels of load were increased by 1 kN until the specimen failed. The stress levels of levels 1~6 were 5, 5.5, 6, 6.5, 7, and 7.5 MPa, respectively. The loading rate was 10 N/s. The computer automatically performed data acquisition during the test, with a sampling interval of 0.01 min during loading and a sampling interval of 5 min after loading stabilization. The last level of load duration was determined by the damage of the test, which was about 7 h for this test, and the remaining levels of load duration were controlled at about 24 h.

The main steps of the rheological test were:

(1) Wrap the dried coal sample with a rubber film (to prevent the fragments from falling apart after crushing, and to keep the original state of the test at the time of failure), and install the LVDT extensometer to measure the axial and lateral deformation;
(2) Put the prepared specimen into the testing machine and adjust the center position of the sample so that the axis of the coal sample coincides with the loading center line of the testing machine to avoid eccentric pressure on the coal sample;
(3) Fix the coal sample with a slight axial stress, and then apply axial stress and hoop stress (5 MPa) to a predetermined value in sequence. The first stage of stress is applied to the coal sample by the servo system, and the system automatically records the deformation value of the sample at that level of stress at the specified time interval. When the specified observation time is reached (the displacement under constant load is less than 0.001 mm/h), it enters the next level of stress test. The coal sample undergoes rheological failure under the action of the last stage of load;
(4) Take out the coal sample, describe its failure mode, and sort out the test data.

## 3. Test Results and Discussion

### 3.1. Typical Creep Curve

**Figure 3:**
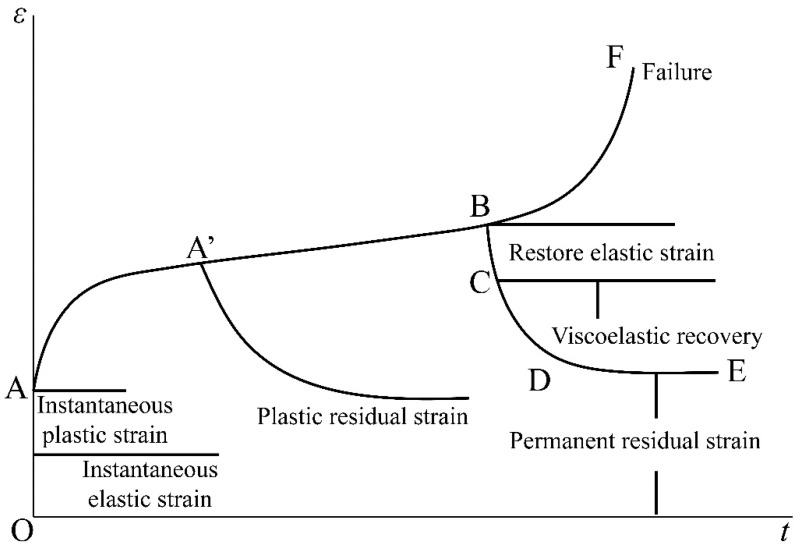
Typical creep curve.

Creep behavior of the coal or rock body is strongly related to the stress state, and the creep process can proceed in a decelerated mode at low-stress levels. Relatively, when the stress level is high, the creep process may proceed in an accelerated mode until it enters the damage state, and the higher the load, the faster the coal or rock damage [30]. The first case mentioned above is called the attenuated creep process, and the second case is called the non-attenuated creep process. The typical creep curve of coal or rock under long-term load is shown in Figure 3.

Among them, the O–A section corresponds to the short-term deformation response of the surrounding rock, and falls under the category of content linked to the stability evaluation of the roadway during the construction period. This part of the deformation consists of both elastic and plastic deformation of the short-term behavior, which is commonly analyzed using the statics approach, and it can be assumed that the material’s strain rate tends to infinity at t = 0. With the extension of time, the material enters the initial creep stage known as A–A’ section. The typical characteristic of this stage is the transition of creep speed from infinite value to finite value. The creep rate in the A’–B section is kept as a constant, known as the stable creep stage, and if it is unloaded at any point of time during this stage, there will be a large permanent residual deformation. After the deformation reaches point B, it will gradually accelerate and reach a certain point F, where sudden damage (creep fracture) may occur. The B–F section is called the accelerated creep stage.

### 3.2. Analysis of Test Results

**Figure 4:**
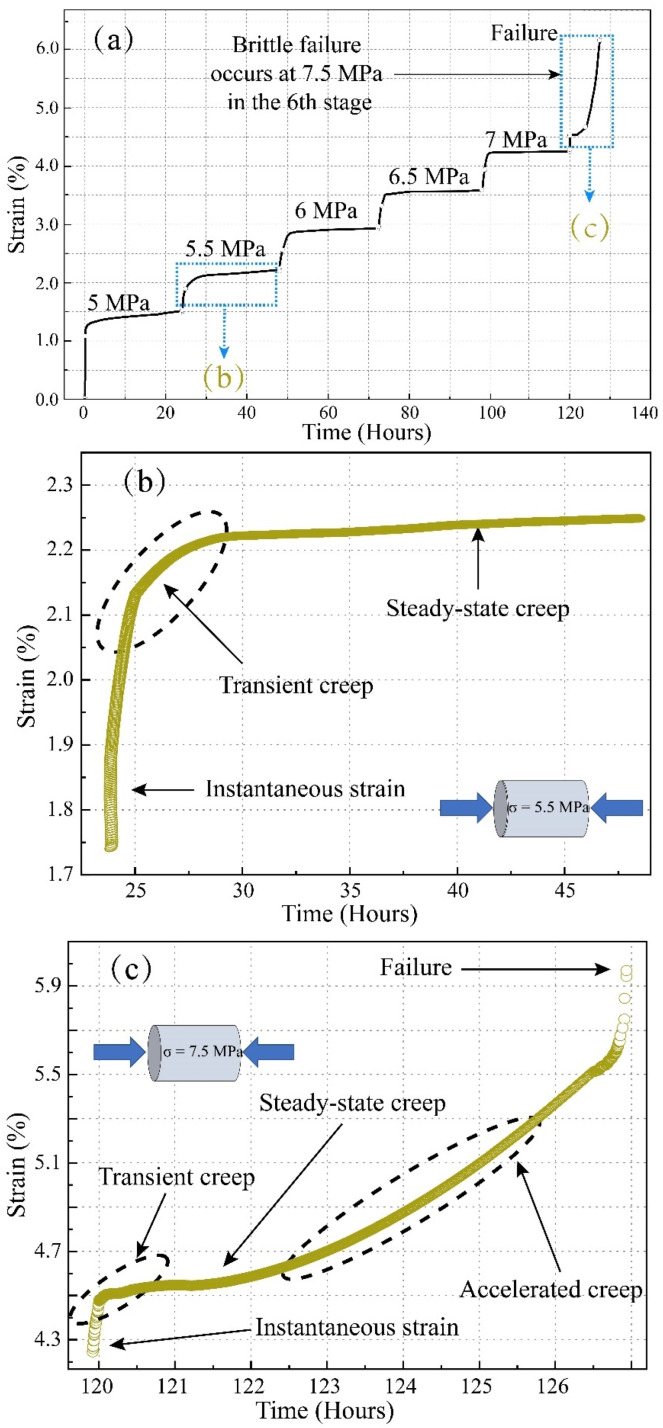
Strain–time curve under multi-stage constant load: (**a**) Creep process curve under multi-stage constant load; (**b**) Stable creep curve under constant load of 5.5 MPa; (**c**) Accelerated creep curve under constant load of 7.5 MPa.

The full-process curve of the axial strain obtained from the test under the multi-stage constant load is shown in Figure 4a below. We applied six level loads on the sample (i.e., 5 MPa, 5.5 MPa, 6 MPa, 6.5 MPa, 7 MPa, and 7.5 MPa). The initial stage of loading was mainly instantaneous strain and transient creep. As the pressure stabilized, attenuated creep was mainly manifested under constant load conditions, as shown in Figure 4b. In the final stage of the load (7.5 MPa constant load), the sample suddenly destabilized and failed, and an accelerated creep curve was obtained, or non-attenuated creep, as shown in Figure 4c.

The test results show that at each level of loading, a transient linear strain was generated, which was the amount of strain from the beginning of loading to the completion of loading. The ratio of the instantaneous strain to the total strain under each load level gradually decreased with the increase in the load level. During the constant load, after 24 h, the axial strain tended to stabilize with deformations of about 0.1 mm for the level 1 load, 0.15 mm for level 2, 0.21 mm for level 3, 0.33 mm for level 4, and 0.71 mm for level 5. The creep curve showed a decaying trend, and the creep rate at all levels of loading decreased to 0 with time, as shown in Figure 4. The creep strain rate under different constant stress gradients shows a power exponential change trend with time, as follows:

(1) $$\varepsilon_{t}=A{\cdot e}^{B\cdot t}$$

**Figure 5:**
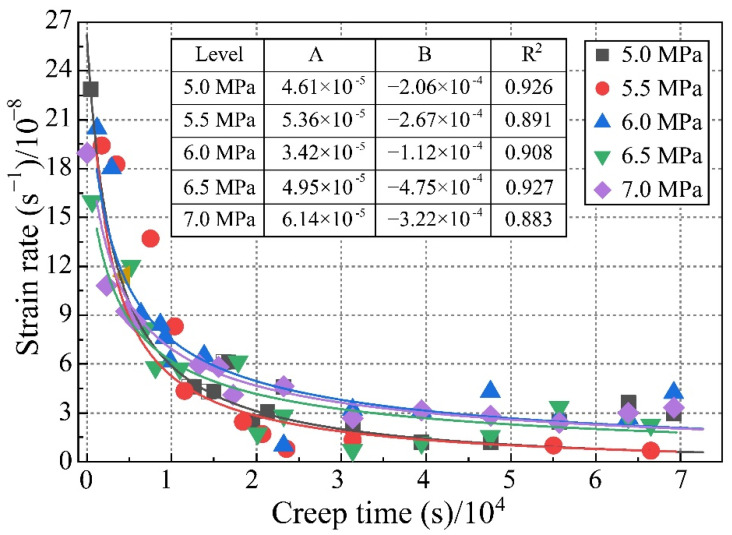
Strain rate curve and fitting results.

In the formula, *A* and *B* are regression analysis parameters, and the fitness values for each stage are given in Figure 5.

To summarize, when a coal body deforms over time owing to a long-term load, its strength characteristics will fluctuate due to the coal body’s plastic deformation features. Plastic elements with varying strength properties can be incorporated to more properly assess coal creep behavior.

### 3.3. Analysis of Rheological Characteristics

**Figure 6:**
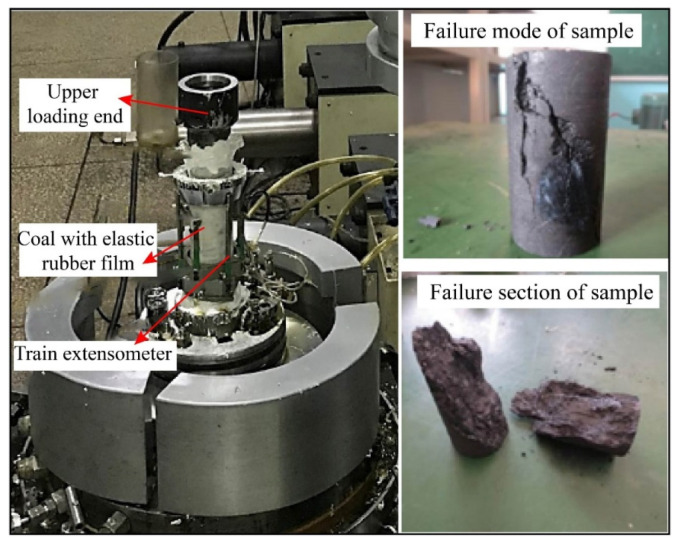
Failure modes of coal samples in triaxial rheological test.

The coal sample was damaged after 127 h of long-term creep deformation, with continuous deformation curves occurring during the whole creep process, which had a significant time effects and nonlinear characteristics. At the beginning of the test (0–5 MPa), the coal sample underwent instantaneous elastic deformation at the moment of loading. With the movement of rock particles, the original open structural surfaces or micro-cracks in the sample were compressed and closed, and the strain on the sample increased rapidly in a short period of time. Subsequently, under low stress and a constant load (5 MPa), the strain of the sample gradually increased, but the deformation rate gradually became flat. At this stage, the micro-element body stayed intact, and the sample underwent viscoelastic deformation; under medium-high stress and a constant load (5.5–7 MPa), the open structural surface and micro-cracks of the sample were further compressed and closed, and the creep rate of the sample also gradually decreased. However, due to the high stress, some of the micro-element bodies underwent plastic failure, and the constant creep rate of the sample was not 0, and at this time, the rock underwent viscoelastic-plastic deformation; at a high stress level (7.5 MPa), the creep rate of the sample decreased continuously after instantaneous elastic deformation. After 0.1 h, it entered the steady-state creep stage and maintained the steady-state rate for 7 h, and then accelerated creep failure occurred. At this stage, the plastic strain of the sample kept accumulating, and the internal pores and cracks expanded and developed, which ultimately led to the destruction of the specimen by penetration. In the rheological experiment, the final failure form of the coal sample is shown in the Figure 6.

The fracture mode of coal sample was mainly shear failure, accompanied by a large degree of local tensile failure. The coal sample had a main shear fracture surface extending from the upper end to the lower end, which divided the rock sample into basically the same two parts. The angle between the main shear fracture surface and the maximum principal stress action surface was approximately 18°, and there were branch small cracks on the main crack. The shear fracture surface was relatively rough, with many fine powders attached to the shear fracture surface, and the fracture slip trace was obvious. In addition, there were many tension failure cracks on the surface of the rock sample. The cracks were concentrated on one side of the rock sample and extended from the lower end to the upper middle part of the rock sample. There were large strip fragments peeling off on the surface of the rock sample.

## 4. New Nonlinear Viscoelastic-Plastic Rheological Model Based on Fractional Calculus

The creep mode is not only time-related, but also has a direct relationship with the applied stress. The classic Burgers model has been proved to be unsatisfactory in describing the nonlinear acceleration characteristics and time effects in the creep process [31]. Therefore, it is necessary to introduce fractional-order elements, nonlinear elements, etc. to replace the linear elements in the model and create a rheological model with variable parameters, in which the rheological characteristics of the coal and its rheological curve with time are portrayed by the method of the variation of relevant parameters with time.

### 4.1. Fractional Element

In recent years, the application of fractional calculus theory in engineering has gradually increased, and fractional derivatives have also been applied to the study of rock constitutive models, which have achieved positive results [32]. There are various ways of defining the fractional calculus. In general, the Riemann–Liouville fractional calculus is used in describing the rheological properties of rocks, which is defined as [33]: Suppose f is continuous segment by segment on (0, +∞) and can be produced on any finite subinterval of [0, +∞], for *t* > 0, Re(*r*) > 0, then consider Equation (2) as the Riemann–Liouville fractional integral of function *f*(*t*) of *r* order.

(2) Jtr0R=d−r[f(t)]dt−r=1Γ(r)∫0t(t−ξ)r−1f(ξ)dξ

where, *R* denotes Riemann–Liouville theory, *J* is the fractional order operator, 0 and *t* are the upper and lower limits of integration, respectively, *r* is the order of fractional integration, and $\Gamma (r)=\int_{0}^{\infty }t^{r-1}e^{-t}dt$ is the Gamma function.

**Figure 7:**
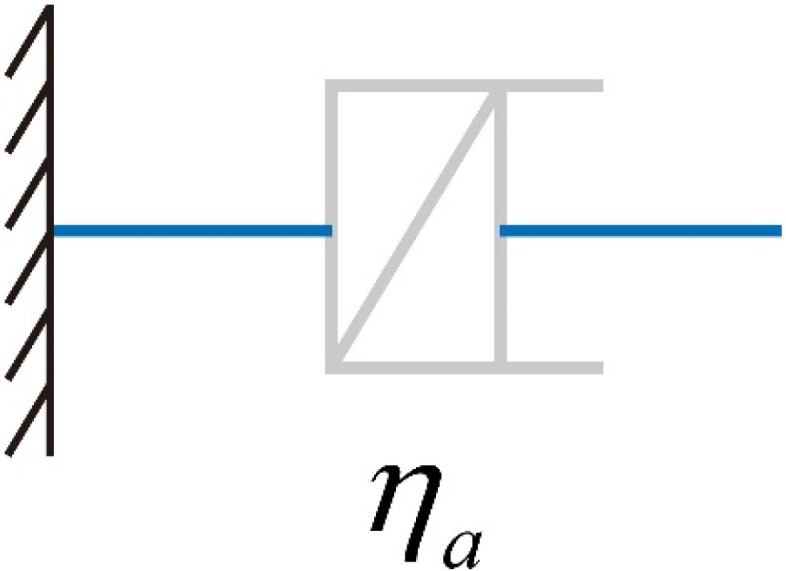
Abel fractional element.

The fractional element (Figure 7) is a variable parameter viscous system consisting of a piston and an Abel kernel, whose main feature is to complement the fact that the integer derivative does not well describe the intermediate development process of the system function. Its value is not only closely related to the state of the current moment, but also to the entire course of history. Compared with Newton’s viscous pot, the distinguishing feature of the fractional element is that its stress–strain relationship is nonlinear. Following the above definition of fractional derivatives, the basic constitutive relationship for Abel viscous pots is easily given by the stress–strain relationship for Newton’s viscous pot:

(3) $$\sigma =\eta_{a}D^{r}\varepsilon (t)=\eta_{a}\frac{d^{r}\varepsilon (t)}{dt^{r}}(0<r<1)$$

where, $\eta_{a}$ is the Abelian viscosity coefficient, *D* is the fractional order operator, and *r* is the fractional order derivative order.

When *r* = 1 in the above Equation (3), it is the integer derivative, and its constitutive relation is the Newtonian viscous pot constitutive relation. Equations (2) and (3) are combined to give a general expression for the constitutive relationship of the Abel viscous pot element (Fractional element):

(4) $$\varepsilon (t)=\frac{\sigma }{\eta_{a}}\frac{t^{r}}{\Gamma (r+1)}$$

### 4.2. Nonlinear Viscoplastic Element

**Figure 8:**
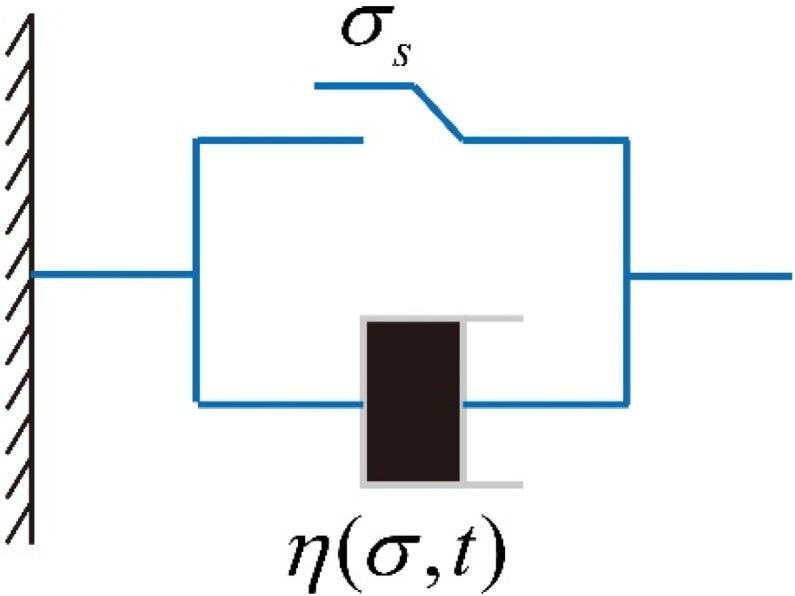
Nonlinear viscous-plastic element.

In this paper, by referring to a large amount of literature on nonlinear viscous-plastic elements [34], the nonlinear viscous-plastic element (Figure 8) is introduced for the accelerated creep stage, and the expression of the viscous coefficient is:

(5) $$\eta (\sigma ,t)=\eta_{0}e^{\frac{-(\sigma -\sigma_{s})}{b}t},(\sigma -\sigma_{s})=\left\{\begin{array}{c} 0,\sigma \leq \sigma_{s}\\ \sigma -\sigma_{s},\sigma >\sigma_{s} \end{array}\right\}$$

where $\eta_{0}$ is the initial viscosity coefficient, $\sigma_{s}$ is the yield stress, *b* is the material constant in MPa·h, and *t* is the loading time.

When $\sigma \leq \sigma_{s}$, then $\eta (\sigma ,t)=\eta_{0}$; at this point, the model cannot slide because it is a plastic element and does not produce deformation, which can be ignored when this model is connected in series with other models.

When $\sigma >\sigma_{s}$, and stress is constant, then $\eta (\sigma ,t)=\eta_{0}e^{\frac{-(\sigma -\sigma_{s})}{b}t}$, and the nonlinear viscosity coefficient η(σ,t) decreases with increasing time *t*. At this point, the component rheology equation is:

(6) $$\dot{\varepsilon }=\frac{\sigma -\sigma_{s}}{\eta (\sigma ,t)}=\frac{\sigma -\sigma_{s}}{\eta_{0}}e^{\frac{\sigma -\sigma_{s}}{b}t}$$

When the stress is constant, the creep equation can be obtained by integrating both sides:

(7) $$\varepsilon =\frac{b}{\eta_{0}}[e^{\frac{\left(\sigma -\sigma_{s}\right)}{b}t}-1]$$

### 4.3. New Fractional Nonlinear Viscoelastic-Plastic Rheological Model

**Figure 9:**
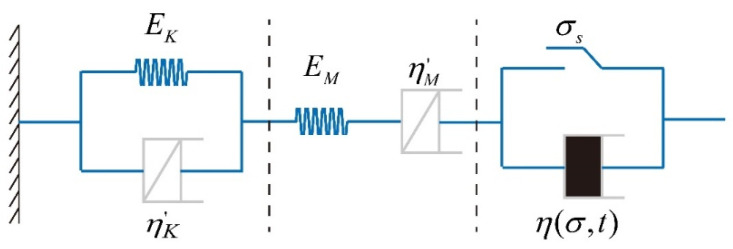
A new fractional nonlinear viscoelastic-plastic model.

In review, the fractional elements are utilized to replace the relevant viscous pots in the Burgers model, and the nonlinear viscous-plastic elements are series connected to construct a new six-element NFNVRM, as shown in Figure 9. The new model is able to reflect not only the nonlinear changes in the decay and stabilization stages of creep, but also the whole process of the accelerated rheological stage.

On each element in series, the stress is equal, and the strain is equal to the sum of strain on each element; on each element in parallel, the strain is equal, and the stress is equal to the sum of stress on each element. So:

(8) $$\{\begin{array}{c} \sigma =\sigma_{M}=\sigma_{K}=\sigma_{N}\\ \varepsilon =\varepsilon_{M}+\varepsilon_{K}+\varepsilon_{N}\\ \dot{\varepsilon }={\dot{\varepsilon }}_{M}+{\dot{\varepsilon }}_{K}+{\dot{\varepsilon }}_{N}\\ \ddot{\varepsilon }={\ddot{\varepsilon }}_{M}+{\ddot{\varepsilon }}_{K}+{\ddot{\varepsilon }}_{N} \end{array}$$

The viscosity coefficients of the two sticky pots in the model both change with time, and the changing law is exponentially changing with time. The one-dimensional constitutive relationship of the new model is as follows:

(9) $$\varepsilon (t)=\frac{\sigma }{E_{M}}+\frac{\sigma }{E_{K}}(1-e^{\frac{-E_{K}}{\eta_{K}}t^{1-\beta }})+\frac{\sigma }{\eta_{a}}\frac{t^{r}}{\Gamma (1+r)}+\frac{b}{\eta_{0}}(e^{\frac{\sigma -\sigma_{s}}{b}t}-1)$$

(1) When $\sigma \leq \sigma_{s}$, the model degenerates to a Burgers model with fractional derivatives, and its creep equation is:
  (10) $$\varepsilon (t)=\frac{\sigma }{E_{M}}+\frac{\sigma }{E_{K}}(1-e^{\frac{-E_{K}}{\eta_{K}}t^{1-\beta }})+\frac{\sigma }{\eta_{a}}\frac{t^{r}}{\Gamma (1+r)}$$
  where β, *r* is the fractional derivative, and $\eta_{a}$ is the initial viscosity coefficient of the Abel viscous pot.

This is such that the Kelvin element viscous pot viscosity coefficient follows the form $\eta_{K}^{\prime }=\eta_{K}t^{\beta }$ and the Maxwell element viscous pot viscosity coefficient follows the form $\eta_{M}^{\prime }=Ht^{1-r}$. Thus, the fractional Burgers model can be written as an expression for the Burgers model, as follows:

(11) $$\varepsilon =\frac{\sigma }{E_{M}}+\frac{\sigma }{\eta_{M}^{\prime }}t+\frac{\sigma }{E_{K}}(1-e^{\frac{-E_{K}}{\eta_{K}^{\prime }}t})$$

**Figure 10:**
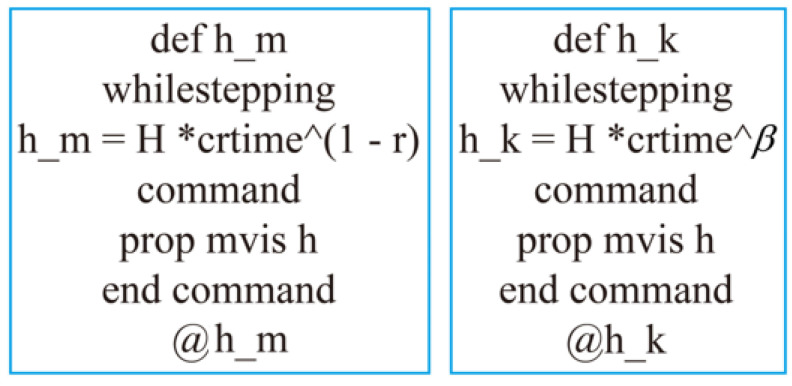
FISH language for implementing fractional models in FLAC3D.

Following this feature, the fractional derivative relation is enabled by C++ programming. That is, instead of modifying the constitutive model in the source program, there is only a need to modify the FLAC3D built-in Burger model source program appropriately to ensure that the viscosity coefficients change over time according to the above-mentioned law. The FISH command is shown in Figure 10.

Following previous experience [35], when the one-dimensional creep model is extended to a three-dimensional creep model, all that is required is to swap the corresponding parameters of the rheological constitutive equation, i.e., $\varepsilon \to e_{ij}$, $\sigma \to S_{ij}$, E→2G, η→2H. The computational relations of the new model are identical to those of the Burgers model, and the following three-dimensional constitutive relations are given directly:

(12) $$\varepsilon (t)=\frac{2\sigma_{2}}{9K}+\frac{\sigma_{1}}{3G_{M}}+\frac{\sigma_{1}}{3G_{K}}(1-e^{\frac{-G_{k}}{\eta_{K}t^{\beta }}t})+\frac{\sigma_{1}}{3Ht^{1-r}}t$$

(2) When $\sigma >\sigma_{s}$, the creep equation is:
  (13) $$\varepsilon =\frac{\sigma }{E_{M}}+\frac{\sigma }{\eta_{M}^{\prime }}t+\frac{\sigma }{E_{K}}(1-e^{\frac{-E_{K}}{\eta_{K}^{\prime }}t})+\frac{b}{\eta_{0}}(e^{\frac{\sigma -\sigma_{s}}{b}t}-1)$$
  where $\frac{\sigma }{E_{M}}$ is the instantaneous elastic strain; $\frac{\sigma }{\eta_{M}^{\prime }}t+\frac{b}{\eta_{0}}(e^{\frac{\sigma -\sigma_{s}}{b}t}-1)$ is the irrecoverable strain, which is viscous flow; and $\frac{\sigma }{E_{K}}(1-e^{\frac{-E_{K}}{\eta_{K}^{\prime }}t})$ is the recoverable strain.

For Maxwell:

$$\begin{array}{c} {\dot{\varepsilon }}_{M}=\frac{{\dot{\sigma }}_{M}}{E_{M}}+\frac{\sigma_{M}}{\eta_{M}^{\prime }}\\ {\ddot{\varepsilon }}_{M}=\frac{{\ddot{\sigma }}_{M}}{E_{M}}+\frac{{\dot{\sigma }}_{M}}{\eta_{M}^{\prime }} \end{array}$$

For Kelvin:

$$\begin{array}{c} \sigma_{K}=E\varepsilon_{K}+\eta_{K}^{\prime }{\dot{\varepsilon }}_{K}\\ {\dot{\sigma }}_{K}=E{\dot{\varepsilon }}_{K}+\eta_{K}^{\prime }{\ddot{\varepsilon }}_{K} \end{array}$$

For Nonlinear body:

$$\begin{array}{c} {\dot{\varepsilon }}_{N}=\frac{\sigma -\sigma_{s}}{\eta_{0}}\exp(\frac{\sigma -\sigma_{s}}{b}t)\\ {\ddot{\varepsilon }}_{N}=\frac{1}{\eta_{0}}(1+\frac{\sigma -\sigma_{s}}{b})\times \exp(\frac{\sigma -\sigma_{s}}{b}t)\times \dot{\sigma }+\frac{{\left(\sigma -\sigma_{s}\right)}^{2}}{b\eta_{0}}\times \exp(\frac{\sigma -\sigma_{s}}{b}t) \end{array}$$

So the constitutive equation of the nonlinear rheological model is:

(14) $$\ddot{\varepsilon }=\frac{\ddot{\sigma }}{E_{M}}+\frac{\dot{\sigma }}{\eta_{M}^{\prime }}+\frac{\dot{\sigma }}{\eta_{K}^{\prime }}-\frac{E_{K}}{\eta_{K}^{\prime }}{\dot{\varepsilon }}_{K}+\frac{E_{K}}{\eta_{K}^{\prime }E_{M}}\dot{\sigma }+\frac{E_{K}}{\eta_{K}^{\prime }\eta_{M}^{\prime }}\sigma +\frac{E_{K}}{\eta_{K}^{\prime }}{\dot{\varepsilon }}_{N}+{\ddot{\varepsilon }}_{N}$$

When sorted out, it becomes:

(15) $$\begin{array}{l} \ddot{\varepsilon }+\frac{E_{K}}{\eta_{K}^{\prime }}\dot{\varepsilon }=\frac{\ddot{\sigma }}{E_{M}}+\left[\frac{1}{\eta_{M}^{\prime }}+\frac{1}{\eta_{K}^{\prime }}+\frac{E_{K}}{\eta_{K}^{\prime }E_{M}}+\frac{1}{\eta_{0}}(1+\frac{\sigma -\sigma_{s}}{b})\times \exp(\frac{\sigma -\sigma_{s}}{b}t)\right]\dot{\sigma }\\ +\frac{E_{K}}{\eta_{K}^{\prime }\eta_{M}^{\prime }}\sigma +\frac{E_{K}}{\eta_{K}^{\prime }\eta_{0}}(\sigma -\sigma_{S})\exp(\frac{\sigma -\sigma_{s}}{b}t)+\frac{{\left(\sigma -\sigma_{s}\right)}^{2}}{b\eta_{0}}\times \exp(\frac{\sigma -\sigma_{s}}{b}t) \end{array}$$

where σ, $\dot{\sigma }$, $\ddot{\sigma }$, ε, $\dot{\varepsilon }$, $\ddot{\varepsilon }$ represent stress, strain and the first-order and second-order derivatives of stress and strain with respect to time; *E*, $\eta^{\prime }$, $\eta_{0}$, *b* denote the modulus of elasticity, viscosity coefficient, initial viscosity coefficient, and material constant; $\sigma_{s}$ indicates the yield stress of the coal rock; and the angular scales M, K, and N denote the corresponding parameters of fractional-order Maxwell, fractional-order Kelvin, and nonlinear viscous-plastic bodies, respectively.

The three-dimensional constitutive relation of the viscous-plastic model is [36,37,38]:

(16) $${\dot{\varepsilon }}_{ij}=\frac{1}{2H}\langle \phi \left(\frac{F}{F_{0}}\right)\rangle \frac{\partial Q}{\partial \sigma_{ij}},\langle \phi \left(\frac{F}{F_{0}}\right)\rangle =\{\begin{array}{c} 0,F<0\\ \phi \left(\frac{F}{F_{0}}\right),F\geq 0 \end{array}$$

where the function ϕ is generally a power function or exponential function; *F* is the rock yield function, according to different yield criteria using different forms; *F*_0_ is the initial reference value of the rock yield function, which is usually taken to be *F*_0_ = 1; *Q* is the plastic potential function, *Q = F* is the associated flow law in plasticity theory, and *Q ≠ F* is the non-associated flow law.

By combining Equations (15) and (16), the three-dimensional rheological equation corresponding to the three-dimensional form of the yield condition *F* > 0 is:

(17) $$\begin{array}{l} {\ddot{e}}_{ij}+\frac{G_{K}}{H_{k}}{\dot{e}}_{ij}=\frac{{\ddot{S}}_{ij}}{E_{M}}+\left[\frac{1}{2H_{M}}+\frac{1}{2H_{k}}+\frac{G_{K}}{2H_{k}H_{M}}+\frac{1}{2H_{0}}\left(1+{\left(\frac{F}{F_{0}}\right)}^{m}\frac{\partial F}{\partial \sigma_{ij}}\frac{t}{b}\right)\times \exp\left({\left(\frac{F}{F_{0}}\right)}^{m}\frac{\partial F}{\partial \sigma_{ij}}\frac{t}{b}\right)\right]{\dot{S}}_{ij}\\ +\frac{G_{K}}{2H_{k}H_{M}}S_{ij}+\frac{G_{K}}{2G_{k}H_{0}}{\left(\frac{F}{F_{0}}\right)}^{m}\frac{\partial F}{\partial \sigma_{ij}}\exp\left({\left(\frac{F}{F_{0}}\right)}^{m}\frac{\partial F}{\partial \sigma_{ij}}\frac{t}{b}\right)+\frac{{\left({\left(\frac{F}{F_{0}}\right)}^{m}\frac{\partial F}{\partial \sigma_{ij}}\right)}^{2}}{2bH_{0}}\times \exp\left({\left(\frac{F}{F_{0}}\right)}^{m}\frac{\partial F}{\partial \sigma_{ij}}\frac{t}{b}\right) \end{array}$$

where $S_{ij}$, ${\dot{S}}_{ij}$, ${\ddot{S}}_{ij}$, ${\dot{e}}_{ij}$, ${\ddot{e}}_{ij}$ represent partial stress, partial strain and the first- and second-order derivatives of partial stress and partial strain with respect to time; and *G* and *H* represent the three-dimensional shear modulus and three-dimensional viscosity coefficient.

When *t* = 0, apply a constant strain $\varepsilon_{c}$, then $\dot{\varepsilon }=0$, $\ddot{\varepsilon }=0$, and the initial stress is $\sigma_{0}=E_{M}\varepsilon_{c}$. The relaxation equation can be obtained from the solution of the rheological equation:

(18) $$\begin{array}{l} \frac{\ddot{\sigma }}{E_{M}}+\left[\frac{1}{\eta_{M}}+\frac{1}{\eta_{k}}+\frac{E_{K}}{\eta_{k}E_{M}}+\frac{1}{\eta_{0}}(1+\frac{\sigma -\sigma_{s}}{b})\times \exp(\frac{\sigma -\sigma_{s}}{b}t)\right]\dot{\sigma }\\ +\frac{E_{K}}{\eta_{k}\eta_{M}}\sigma +\frac{E_{K}}{\eta_{k}\eta_{0}}(\sigma -\sigma_{S})\exp(\frac{\sigma -\sigma_{s}}{b}t)+\frac{{\left(\sigma -\sigma_{s}\right)}^{2}}{b\eta_{0}}\times \exp(\frac{\sigma -\sigma_{s}}{b}t)=0 \end{array}$$

When $t=t_{c}$, Then $\dot{\sigma }=0$, $\ddot{\sigma }=0$, and the unloading equation is obtained by substituting the rheological equation. From the rheological equations, the unloading equations of the fractional Burgers model and the nonlinear viscous-plastic body can be superimposed to obtain the unloading equations of the model.

For the fractional Burgers model:

(19) $$\varepsilon =\frac{\sigma }{E_{M}}t_{c}+\frac{\sigma }{E_{K}}(1-e^{\frac{-E_{K}}{\eta_{K}^{\prime }}t})e^{\frac{E_{K}}{\eta_{K}^{\prime }}\left(t_{c}-1\right)}$$

For the Nonlinear viscous-plastics:

(20) $$\varepsilon_{N}=\frac{b}{\eta_{0}}\left[\exp(\frac{\sigma -\sigma_{s}}{b}t_{c})-1\right]$$

Therefore, by superimposing Equations (19) and (20), the new model unloading equation is obtained as:

(21) $$\varepsilon =\frac{\sigma }{E_{M}}t_{c}+\frac{\sigma }{E_{K}}(1-e^{\frac{-E_{K}}{\eta_{K}^{\prime }}t})e^{\frac{E_{K}}{\eta_{K}^{\prime }}\left(t_{c}-1\right)}+\frac{b}{\eta_{0}}\left[\exp(\frac{\sigma -\sigma_{s}}{b}t_{c})-1\right]$$

### 4.4. Model Parameter Identification

**Figure 11:**
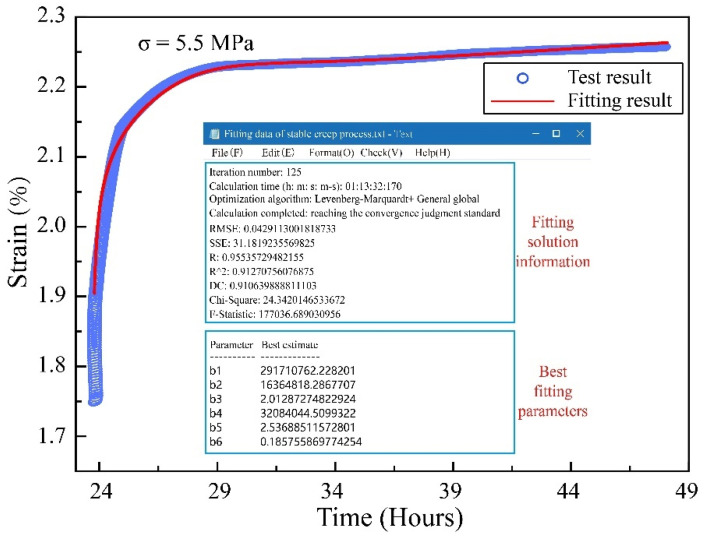
Fitting results of stable creep process.

**Figure 12:**
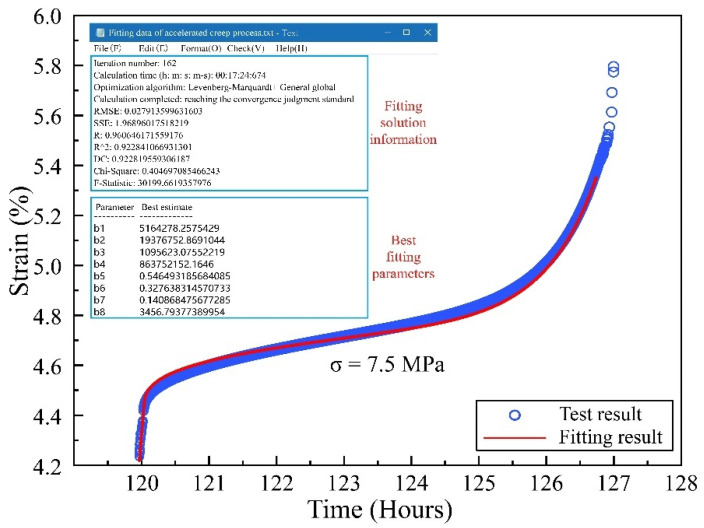
Fitting results of accelerated creep process.

The complete model proposed in this paper contains eight parameters, which can accurately reflect the nonlinear creep characteristics of coal. Combined with the experimental data, a nonlinear fitting procedure was written for NFNVRM fitting and parameter identification using 1STOP software (1STOP, Redfern, NSW, Australia) based on McCourt’s algorithm. To simplify the calculation, let $E_{M}$ = *b1*, $E_{K}$ = *b2*, $\eta_{K}$ = *b3*, $\eta_{a}$ = *b4*, β = *b5*, *r* = *b6*, $\eta_{0}$ = *b7*, *b* = *b8* to pick the two typical stages of decay creep (5.5 MPa) and accelerated creep (7.5 MPa) for the fitting. The fitting results are as follows (Figure 11 and Figure 12).

According to the fitting results, the NFNVRM established in this paper can accurately fit the accelerated creep process and nonlinear asymptotic characteristics of coal. Besides, the fitted correlation coefficient r reached 0.9606, which verified the correctness and applicability of the model.

## 5. Secondary Development Based on Burgers Program Code

FLAC3D software provides a convenient secondary development interface, which is easier than other finite element programs, and the execution efficiency of user-defined models is the same as the models included in the program. In this paper, FLAC3D was used to develop the coal nonlinear rheology numerical program, which not only ensured the computational efficiency, but also wrote relatively less code.

### 5.1. New Model Calculation Principle

The constitutive equations of the model in FLAC3D and the step-size solutions of all motion equations are calculated by displaying the finite difference format. The constitutive equation is derived from the definition of basic stress, strain, and Hooke’s law. And the motion balance equation directly applies Cauchy’s equation of motion, which is derived from Newton’s law of motion. During calculation, the equations of motion are first invoked, and the new velocity and displacement are calculated from the initial stress and boundary conditions, followed by calculating the strain rate from the velocity, and then the new stress or force is obtained according to the constitutive equation. The calculation principle of the finite difference method is shown as follows.

According to Figure 7, the model is divided into 3 parts, namely Maxwell, Kelvin and nonlinear viscous-plastic body. The three-dimensional creep equation of the model is written in the form of the stress and strain deflection increment:

(22) $$\Delta e_{ij}=\Delta e_{ij}^{K}+\Delta e_{ij}^{M}+\Delta e_{ij}^{P}$$

In the formula, the superscripts *K*, *M*, and *P* respectively represent the corresponding quantities of Kelvin elements, Maxwell elements, and nonlinear viscous-plastic elements, and $\Delta e_{ij}$ represent the increase in partial strain.

(1) For Kelvin:
  (23) $$S_{ij}=2H_{K}{\dot{e}}_{ij}^{K}+2G_{K}e_{ij}$$
  where, $e_{ij}$, ${\dot{e}}_{ij}$ denotes the partial strain and the first-order derivatives of partial strain with respect to time, respectively.

The change in unit time is:

(24) $${\bar{S}}_{ij}\Delta t=2H_{K}e_{ij}^{K}+2G_{K}{\bar{e}}_{ij}^{K}\Delta t$$

where the superscripts N and O indicate the new and old values. ${\bar{S}}_{ij}=\frac{S_{ij}^{N}+S_{ij}^{O}}{2}$, ${\bar{e}}_{ij}^{K}=\frac{e_{ij}^{K,N}+e_{ij}^{K,O}}{2}$, $\Delta e_{ij}^{K}=e_{ij}^{K,N}-e_{ij}^{K,O}$,.

When sorted out:

(25) $$e_{ij}^{K,N}=\frac{1}{A}[Be_{ij}^{K,O}+\frac{\Delta t}{4H_{K}}(S_{ij}^{N}+S_{ij}^{O})]$$

where $e_{ij}^{K,O}$, $e_{ij}^{K,N}$, represent the new and old values of Kelvin element bias strain. $A=1+\frac{G_{K}}{2H_{K}}\Delta t$, $B=1-\frac{G_{K}}{2H_{K}}\Delta t$.

(2) For Maxwell:
  (26) $$\Delta e_{ij}^{M}=\frac{\Delta S_{ij}}{2G_{M}}+\frac{{\bar{S}}_{ij}}{2H_{M}}\Delta t$$

(3) For Non-linear viscous-plasticity:

For the usual Mohr–Coulomb yield function, using the associated flow law, *Q = F*. At this point, *F* is a switching function of the following form:

(27) $$F=\frac{1}{3}J_{1}\sin\varphi +\sqrt{J_{2}^{\prime }}\left(\cos\theta -\frac{1}{\sqrt{3}}\sin\theta \sin\varphi \right)-c\cos\varphi$$

where *J*_1_ is the first invariant of stress, $J_{1}=\sigma_{1}+\sigma_{2}+\sigma_{3}$, *B* is the second invariant of stress bias, $J_{2}^{\prime }=\frac{1}{6}\left[{\left(\sigma_{1}-\sigma_{2}\right)}^{2}+{\left(\sigma_{2}-\sigma_{3}\right)}^{2}+{\left(\sigma_{3}-\sigma_{1}\right)}^{2}\right]$, *C* is the angle of internal friction, and *D* is the cohesion.

Expanding the Equation (22) using Taylor’s formula yields:

(28) $$\varepsilon_{ij}^{P}=\frac{b}{2H_{0}}[e^{\langle F\rangle \frac{\partial F}{\partial \sigma_{ij}}\frac{t}{b}}-1]$$

(29) $$\Delta e_{ij}^{P}=\frac{b}{2H_{0}}[e^{\langle F\rangle \frac{\partial F}{\partial \sigma_{ij}}\frac{t}{b}}-1]-\frac{\Delta e_{kk}^{P}}{3}\delta_{ij}$$

where $\Delta e_{kk}^{P}$ denotes the spherical strain bias of a nonlinear viscous-plastic body of the form

$$\Delta e_{kk}^{P}=\frac{b}{2H_{0}}\left[\exp\left(\langle F\rangle \frac{\partial F}{\partial \sigma_{11}}\frac{t}{b}\right)+\exp\left(\langle F\rangle \frac{\partial F}{\partial \sigma_{22}}\frac{t}{b}\right)+\exp\left(\langle F\rangle \frac{\partial F}{\partial \sigma_{33}}\frac{t}{b}\right)-3\right]$$

In accordance with the expressions for the stress–strain increments of the nonlinear viscoelastic-plastic rheological model, the program code was written in VC++6.0 and the new model was embedded into the software through the UDM interface program provided by FLAC3D software.

### 5.2. Key Technologies for Secondary Development

**Figure 13:**
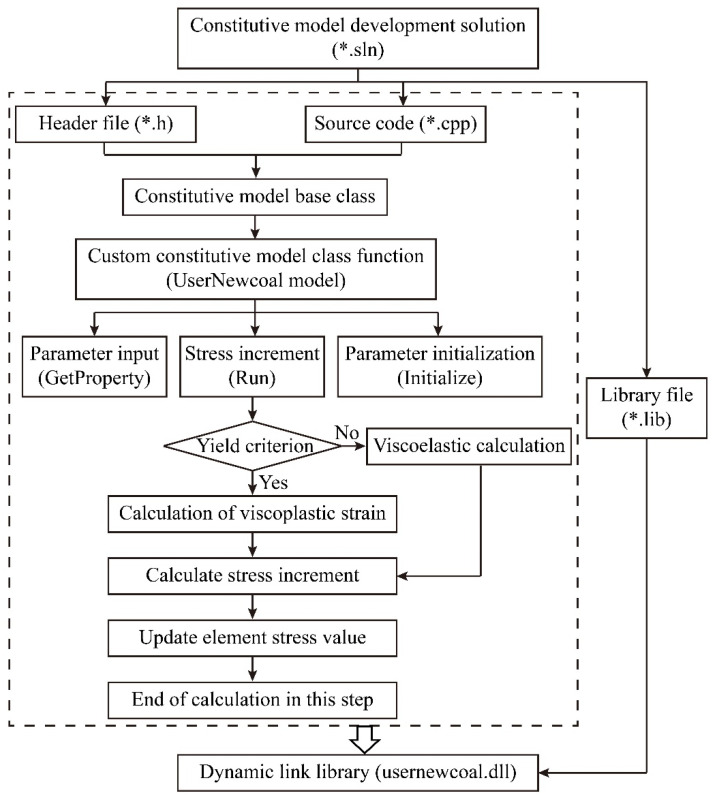
Compilation flowchart.

The secondary development of DLL is done in the program Visual Studio 2010 for the main program to call and execute. The user-defined model in FLAC3D belongs to the same base class (Class Constitutive Model) as the model that comes with the software, and both of them have the same execution efficiency. The flow of secondary development of Flac3D custom constitutive model is shown in Figure 13 below.

**Figure 14:**
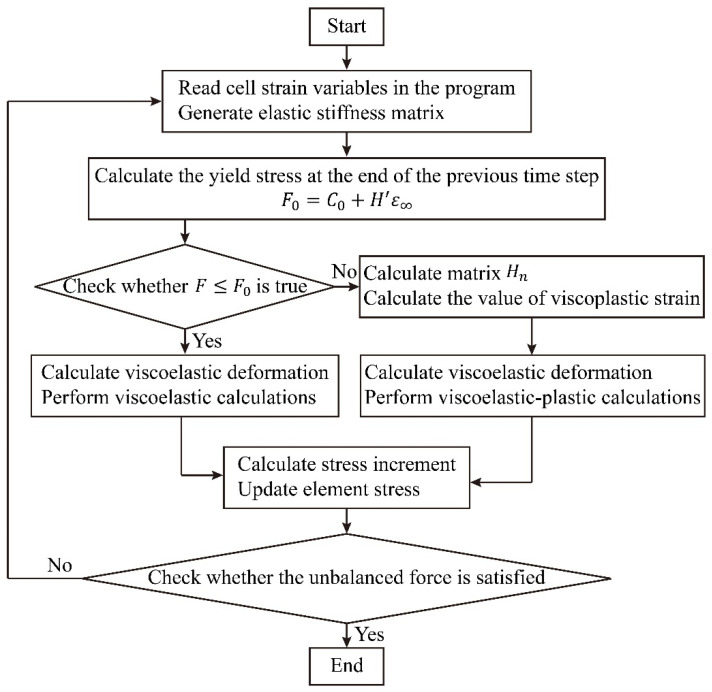
Flowchart of calculation program.

(1) Visual Studio is used to create a solution (*.sln file) to develop the project (*.vcproj). The files shown in Table 2 are included in the UDM file provided with the software.
(2) Add the source and header files for the custom constitutive model. It is not necessary to rewrite all of the code, and the function interfaces in the header and source files do not need to be modified. The user can make changes based on the source code and header files provided with the program. The modifications required are:
  - Modify the registration number of the model;
  - Modify the return name string of the custom constitutive model, which corresponds to the pointer to the string returned when the model is called with the MODEL command in the program;
  - Modify the model name string;
  - The clone function is modified to create a new object of the same class as the current object, returning a pointer to the Constitutive Model type, which must be the same as the name after the EXPORT function;
  - Modification of lateral limit modulus function, bulk modulus function and shear modulus function;
  - The definition of private variables: under the keyword PRIVATE, which defines the direct and indirect variables needed for the constitutive model.
(3) After modifying and regenerating the solution, the DLL file will be generated in the Debug folder in the project folder. The model operations are called in the program for result verification, and the computational flow is shown in Figure 14 below.

### 5.3. Numerical Examples Validated

**Figure 15:**
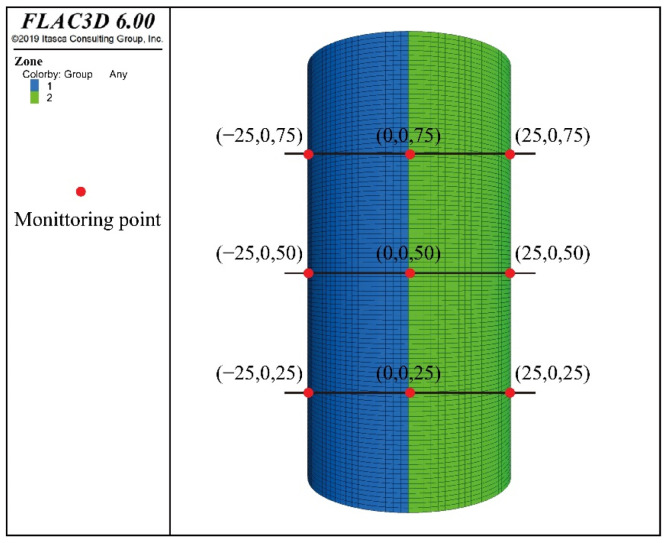
Model and distribution map of monitoring points.

The model size was the same as that of the laboratory creep sample, with a cylindrical diameter of 50 mm and a height of 100 mm, and the cylindrical model was established using the column mesh in the software. In return, the model was divided into 746,375 cells in order to accurately measure the deformation of each part of the model. The vertical load was applied at the top and the perimeter pressure of 5 MPa was applied around the column. The vertical displacement constraint was used at the bottom of the model, without imposing boundary conditions in the lateral direction. A total of nince monitoring points were arranged, and distributed in positions 1/4, 1/2, and 3/4 of the model. The model is shown in Figure 15.

**Figure 16:**
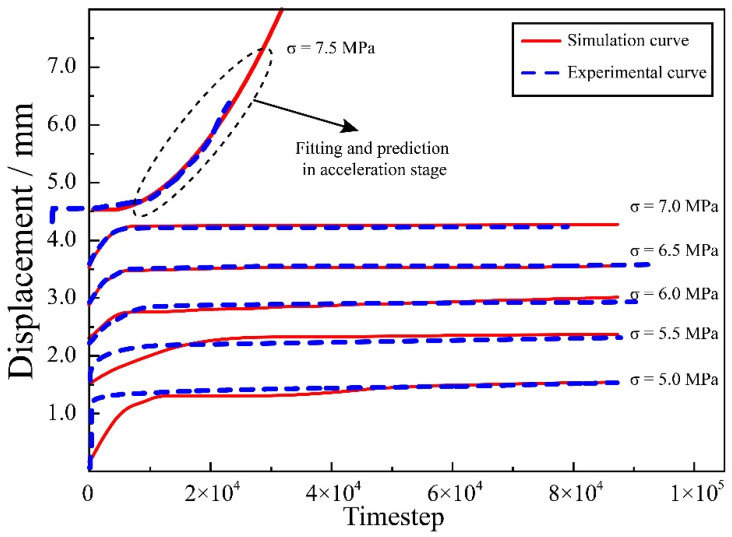
The comparison between experimental curve and simulation curve.

**Figure 17:**
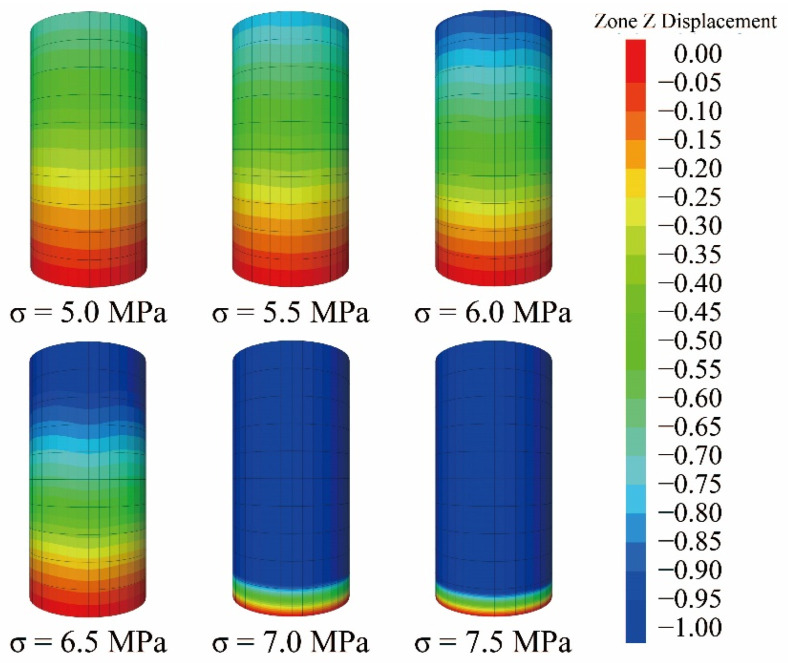
Creep displacement cloud diagram of the whole simulation process.

The calculated parameters were determined according to the results of the triaxial rheological test parameter identification, and the accelerated creep process parameters were taken as the simulated experimental values: $E_{M}$ = 5.164 MPa, $E_{M}$ = 19.377 MPa, $\eta_{K}$ = 1.096 MPa·h, $\eta_{a}$ = 863.752 MPa·h, β = 0.546, *r* = 0.328, $\eta_{0}$ = 0.141 MPa·h, b = 3.457 GPa·h. The loading conditions were the same as those of the indoor tests, and the deviating stresses were 5 MPa, 5.5 MPa, 6 MPa, 6.5 MPa, 7 MPa, and 7.5 MPa, respectively, applied to the model for load levels 1 to 6. The hist write command was used to import the deformation values of the monitoring points into a text file, and the test curves and clouds of the monitoring nodes were calculated based on the numerical calculation results at the selected stress levels. The average values of the nine measurement points were calculated. The comparison between the experimental curve and simulation curve is shown in Figure 16 and displacement nephogram is shown in Figure 17 as follows.

As can be seen from Figure 14, the numerical results were in good agreement with the creep deformation values and variation laws of the indoor creep tests. When the stress level was lower than the yield stress, the axial deformation had two stages of decay creep and steady-state creep, and the strain value eventually tended to stabilize. When the stress level exceeded the yield stress, the creep curve still showed the decay stage and steady-state in the early stage, but in a very short period of time, it went over to the acceleration stage. It can be shown that the program developed by the custom nonlinear model was applicable. However, the creep curves obtained from the numerical simulations had no transient deformation, which is due to the fact that the constitutive model did not contain a transient deformation calculation component.

### 5.4. Creep Prediction of Surrounding Rock

**Figure 18:**
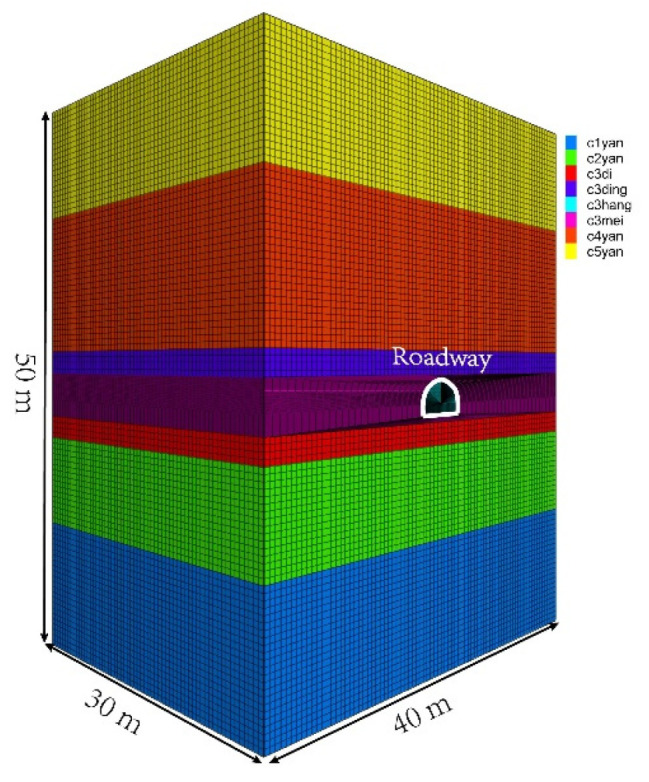
Geometric model of transportation roadway in 2233 working face.

Taking the transportation roadway of a 2233 working face in an S Mine as the research object (See reference [39] for mine overview), the NFNVRM was applied to predict the creep process of the surrounding rock of the roadway. The size of the geometric model was 40 m × 30 m × 50 m (L × W × H), which was divided into 1,218,060 cells, as shown in Figure 18.

The displacement was fixed around and at the bottom of the model, and a static load of 20 MPa was applied at the top to simulate the overburden weight. The model was divided into five layers. The NFNVRM constitutive model was used to study the influence of creep characteristics on the deformation of the roadway and its rock. The Mohr–Coulomb constitutive model was used for the remaining layers to restore the surrounding rock properties of the roadway as much as possible. Through field investigation and sampling, the property parameters of the coal and adjacent rock layers were obtained, as shown in Table 3.

**Figure 19:**
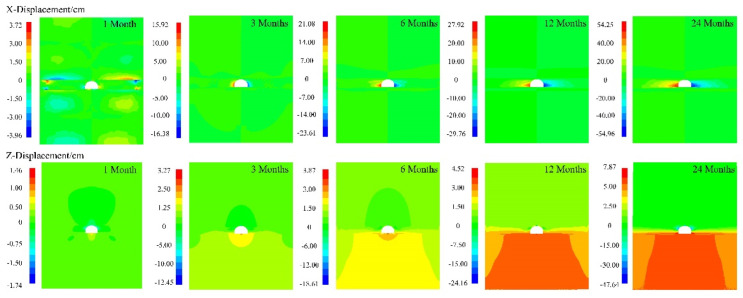
Displacement nephogram of roadway during 24 months of creep.

The creep deformation evolution process of the surrounding rock was long, and took 24 months for analysis and a 1-month interval for calculation. The horizontal and vertical displacement nephogram of representative time points can accurately reflect the site situation, as shown in Figure 19.

From the X-displacement nephogram, it can be seen that the maximum displacement occurred at the arch shoulder of the roadway. The horizontal convergence displacement at 1, 3, 6, 12, and 24 months was 3.96, 16.38, 23.61, 29.76, and 54.96 cm, respectively. In the same time interval, the D-values were 12.42, 7.23, 6.15, and 25.2 cm, respectively. From the Z-displacement nephogram, the settlement of the arch crown and the bulge of the arch bottom existed during the creep period. The maximum displacement occurred at the top of the arch. At 1, 3, 6, 12, and 24 months, the maximum deformation values of the roadway arch crown subsidence were 1.74, 12.45, 18.61, 24.16, and 47.64 cm, respectively. At the same interval in the first year, the D-values were 10.71, 6.16 cm, 5.55 cm, and 23.48 cm, respectively.

In conclusion, at the initial stage of creep, the deformation rate of surrounding rock changed from fast to slow and gradually became stable, and the deformation of the surrounding rock gradually converged. However, due to long-term creep, the coal and rock properties gradually changed from elastic to plastic. If the support is not strengthened in time, the plastic surrounding rock cannot be restored to its original state, and the deformation will continue to increase, which will lead to roadway instability. Finally, it will lead to the occurrence of geological environment disasters such as surface subsidence.

## 6. Conclusions

In this contribution, a triaxle test of rheological properties was carried out on a coal samples from a deep mine roadway. We established a six-element NFNVRM which can simulate the creep characteristics of coal samples throughout the process, based on the analysis of the experimental results. Meanwhile, the constitutive equations of the model were derived, fitted to the test curves, and the parameters of the rheological model of the coal samples in the surrounding rock were obtained. The secondary development of the custom constitutive model was completed by means of FLAC3D finite difference software, which was used to numerically simulate the long-term deformation of coal samples and to verify the accuracy of the model. The main conclusions and results obtained are as follows.

(1) When the stress level is low, the creep rate curve has a decay stage and a steady state stage, and the steady state creep rate tends to increase with the increase of the stress level; When the stress level is higher than the yield threshold, the creep rate curve can appear an acceleration stage after the decay stage and steady stage. During the creep process, the coal sample goes through the stages of crack compaction, elastic deformation, crack expansion, and macro fracture. The instability failure mode of the coal sample mainly consists of shear failure with local tension failure.
(2) The NFNVRM capable of simulating the full process characteristics of coal sample creep was developed by introducing Abel elements and nonlinear viscous-plastic elements. The creep equation, rheological equation, relaxation equation, and unloading equation of the model were also derived. Based on the creep data, 1STOP software was used to identify each parameter of the model, and the fitted curves were in good agreement with the test curves. The NFNVRM provides the theoretical basis for engineering applications.
(3) Based on the interface between FLAC3D software and Microsoft Visual Studio 2010 software (Microsoft Corporation, Redmond, WA, USA), the program of the NFNVRM constitutive model was compiled to generate a dynamic link library file. The program was called to simulate the triaxial compression creep, and the coincidence degree between the simulation curve and the experimental curve was more than 90%, which could well reflect the change of the whole creep process. The validity of the model was verified.
(4) The deformation of the surrounding rock of a transport roadway in an S Mine was predicted for a long time, and the simulation results conformed to the creep law. Based on this, reasonable measures can be taken to avoid casualties and losses. The research results are of great significance for ensuring geological environment safety and social public health.

## Figures and Tables

**Table 1:** Determination results of uniaxial compressive strength of coal samples.

Samples Number	Samples Size Diameter × Height (mm)	Cross-Sectional Area of Samples (cm^2^)	Failure Load (kN)	Compressive Strength (MPa)	Average (MPa)
M-4-1	50.80 × 99.50	20.268	21.93	8.50	9.986
M-4-2	49.34 × 99.92	19.120	18.39	9.62	
M-4-3	49.62 × 100.00	19.338	20.55	10.63	
M-4-4	49.62 × 100.54	19.338	19.51	10.09	
M-4-5	49.22 × 100.48	19.027	21.10	11.09	

**Table 2:** UDM file description table.

File Name	Instruction
Example_src	User Mohr–Coulomb and User Burgers model source code
Axes.h	Defines the coordinate system header file
Conmodel.h	Defines the structure of constitutive model data communication
Contable.h	Defines the table header files needed for the constitutive model
Stensor.h	The header file that defines the tensor
Burgers.dat	An example of using User Burgers
Udm.sln	The solution required for a custom constitutive model
Vcmodels.lib	Custom model required library file
Udm.vcproj	Engineering files required for custom model
Readme.txt	Description file

**Table 3:** Parameter measurement of each coal seam.

**Coal Layer**	**Bulk Modulus/GPa**	**Kelvin** **Shear** **Modulus/GPa**	**Kelvin** **Viscosity/GPa·d**	**Maxwell** **Shear Modulus/MPa**	**Maxwell** **Viscosity/GPa·d**	**Coal Seam** **Thickness/m**	**Density/(kg/m^3^)**
c3mei	10.86	8.6	5.52	14.65	9.58	5	1350
c3ding	19	45	75	30	40	2	2500
c3di	12	25	60	22	20	2	2350
**Rock Layer**	**Bulk** **Modulus/GPa**	**Shear** **Modulus/GPa**	**Internal** **Friction** **Angle/°**	**Cohesion/MPa**	**Tensile Strength/** **MPa**	**Coal Seam** **Thickness/m**	**Density/(kg/m^3^)**
c1yan	21	17.08	34	20.6	6.71	10.5	2300
c2yan	14.65	5.62	32	4.8	1.03	8	2550
c4yan	12.22	9.79	37	5.15	2.6	12.5	2600
c5yan	27.35	24.05	35	26	8.46	10	2450

## Data Availability

Not applicable.
